# Cardiorespiratory fitness attenuates age-associated aggregation of white matter hyperintensities in an at-risk cohort

**DOI:** 10.1186/s13195-018-0429-0

**Published:** 2018-09-24

**Authors:** Clayton J. Vesperman, Vincent Pozorski, Ryan J. Dougherty, Lena L. Law, Elizabeth Boots, Jennifer M. Oh, Catherine L. Gallagher, Cynthia M. Carlsson, Howard A. Rowley, Yue Ma, Barbara B. Bendlin, Sanjay Asthana, Mark A. Sager, Bruce P. Hermann, Sterling C. Johnson, Dane B. Cook, Ozioma C. Okonkwo

**Affiliations:** 1 Geriatric Research Education and Clinical Center, William S. Middleton Memorial Veterans Hospital, Madison, WI 53705 USA; 2 Wisconsin Alzheimer’s Disease Research Center, University of Wisconsin School of Medicine and Public Health, Madison, WI 53792 USA; 3 Wisconsin Alzheimer’s Institute, University of Wisconsin School of Medicine and Public Health, Madison, WI 53705 USA; 4 Department of Kinesiology, University of Wisconsin School of Education, Madison, WI 53792 USA; 5 Department of Neurology, University of Wisconsin School of Medicine and Public Health, Madison, WI 53705 USA; 6 Research Service, William S. Middleton Memorial Veterans Hospital, Madison, WI 53705 USA; 7 Department of Radiology, University of Wisconsin School of Medicine and Public Health, Madison, WI 53705 USA; 8 Department of Psychology, University of Illinois-Chicago, Chicago, IL 60607 USA; 9 Rush Alzheimer’s Disease Center, Rush University Medical Center, Chicago, IL 60612 USA; 10 Department of Medicine and Alzheimer’s Disease Research Center, University of Wisconsin School of Medicine and Public Health, Madison, WI 53792 USA

**Keywords:** Alzheimer’s disease, White matter hyperintensities, Cardiorespiratory fitness

## Abstract

**Background:**

Age is the cardinal risk factor for Alzheimer’s disease (AD), and white matter hyperintensities (WMH), which are more prevalent with increasing age, may contribute to AD. Higher cardiorespiratory fitness (CRF) has been shown to be associated with cognitive health and decreased burden of AD-related brain alterations in older adults. Accordingly, the aim of this study was to determine whether CRF attenuates age-related accumulation of WMH in middle-aged adults at risk for AD.

**Methods:**

One hundred and seven cognitively unimpaired, late-middle-aged adults from the Wisconsin Registry for Alzheimer’s Prevention underwent 3 T magnetic resonance imaging and performed graded maximal treadmill exercise testing from which we calculated the oxygen uptake efficiency slope (OUES) as our measure of CRF. Total WMH were quantified using the Lesion Segmentation Tool and scaled to intracranial volume. Linear regression adjusted for APOE4 carriage, family history, body mass index, systolic blood pressure, and sex was used to examine relationships between age, WMH, and CRF.

**Results:**

As expected, there was a significant association between age and WMH (*p* < .001). Importantly, there was a significant interaction between age and OUES on WMH (*p* = .015). Simple main effects analyses revealed that the effect of age on WMH remained significant in the Low OUES group (*p* < .001) but not in the High OUES group (*p* = .540), indicating that higher CRF attenuates the deleterious age association with WMH.

**Conclusions:**

Higher CRF tempers the adverse effect of age on WMH. This suggests a potential pathway through which increased aerobic fitness facilitates healthy brain aging, especially among individuals at risk for AD.

## Background

With the aging population, the number of people with Alzheimer’s disease (AD) in the United States is projected to reach 13.8 million people by 2050, in the absence of preventative or curative therapies [1]. White matter hyperintensities (WMH), commonly observed in older adults, are characterized by bright areas on magnetic resonance imaging (MRI) using T2-weighted or T2 fluid-attenuated inversion recovery (FLAIR) sequences [16, 47]. WMH have been shown to predict AD earlier in life, and may be the “second hit” required to progress a person to clinical AD [8, 16, 33, 40]. Indeed, WMH are now considered by some to be a core component of AD pathophysiology, and/or caused by chronic ischemia associated with cerebral small vessel disease [9, 33]. The factors that most contribute to the development of WMH are aging and cardiovascular disease [34, 39, 49].

Cardiorespiratory fitness (CRF), an index of habitual physical activity, has been associated with preserved cognitive function and brain structure in older adults [10, 20, 21, 25, 27, 38]. It has also been associated with a lower risk of dementia in the elderly [14, 18, 46]. Interestingly, individuals with higher CRF have also been shown to have lower WMH [7, 13, 37]. This association may indicate that by leading a physically active lifestyle, an individual might slow their accumulation of WMH as they age, and thus enjoy healthier brain aging.

Although peak oxygen consumption (VO_2 peak_) is traditionally regarded as the gold standard measure for CRF [2], older adults as a whole are known to struggle with meeting the criteria for peak effort during maximal graded exercise testing (GXT). The oxygen uptake efficiency slope (OUES) was developed as an effort-independent measure of CRF that is nonetheless highly correlated with VO_2 peak_ [3, 24]. Accordingly, the OUES served as our index of CRF in this study, which examined associations between CRF, age, and WMH [19]. We hypothesized that older age would be associated with more WMH, but that higher CRF would attenuate this deleterious effect of aging on WMH.

## Methods

### Participants

We utilized data provided by 107 participants enrolled in an ancillary study—Fitness, Aging, and the Brain—of the Wisconsin Registry for Alzheimer’s Prevention (WRAP). WRAP is a longitudinal study consisting of approximately 1500, late-middle-aged adults who were free of dementia and were between the ages of 40 and 65 years at study entry [28]. The cohort is enriched with risk factors for AD including positive family history for AD (FH) and/or apolipoprotein E ε4 allele (APOE4) carriage [28, 32, 42]. Participants were enrolled in the ancillary study if they were determined to have no MRI contraindications and could perform a GXT safely. The mean amount of time between the MRI and GXT was 1.04 ± 1.04 years. All study procedures were approved by the University of Wisconsin Institutional Review Board and each participant provided informed consent prior to participation.

### Graded exercise testing

GXT was performed using a modified Balke protocol [4]. A comfortable, yet quick, walking speed was determined for each participant before testing, as a safety measure. For those able to, a walking speed of 3.5 miles per hour was used throughout the test. Every 2 min, the incline of the treadmill was increased by 2.5% until the participant reached volitional exhaustion. Oxygen uptake (VO_2_), carbon dioxide production, minute ventilation (VE), heart rate, and work rate were measured continuously using a metabolic cart and two-way nonrebreathing valve (TrueOne® 2400; Parvomedics, Sandy, UT, USA). The OUES was determined for each participant by calculating the regression slope from the linear relationship of absolute VsO_2_ (ml·min^− 1^) plotted as a function of log_10_ VE (ml·min^− 1^) (i.e., VO_2_ = *a*log_10_VE + *b*) [3]. The OUES values were then adjusted for body surface area (BSA) to account for individual differences [24]. BSA was calculated using the Mosteller formula (BSA = 0.016667 × *W*^0.5^ × *H*^0.5^). A higher OUES value (i.e., a steeper VO_2_ / VE slope) indicates more efficient oxygen extraction from the cardiopulmonary system by the working skeletal muscles [3]. Because the OUES value is calculated as a regression slope, the unit is arbitrary. The OUES computation only included metabolic data collected during the GXT and excluded the warm up and recovery stages due to irregular ventilation that is often observed during those stages. We have previously shown excellent reliability (ICC = .995, *p* < .001) between OUES values calculated at 75%, 90%, and 100% of the exercise duration [19]. Therefore, we used the OUES values that sampled the entire exercise duration (100%) as the primary CRF variable for the current study.

### Brain imaging acquisition

MRI scanning was performed on a GE × 750 3 T scanner (General Electric, Waukesha, WI, USA) with an eight-channel head coil and parallel imaging with the Array Spatial Sensitivity Encoding Technique. A T1-weighted volume scan was acquired in the axial plane with a 3D fast spoiled gradient-echo sequence using the following parameters: inversion time (TI) = 450 ms; repetition time (TR) = 8.2 ms; echo time (TE) = 3.2 ms; flip angle = 12°; acquisition matrix = 256 mm × 256 mm, field of view (FOV) = 256 mm; slice thickness = 1.0 mm, no gap, yielding a voxel resolution of 1 mm isometric. A 3D T2 FLAIR sequence was acquired in the sagittal plane using the following parameters: TI = 1868 ms; TR = 6000 ms; TE = 123 ms; flip angle = 90°; acquisition matrix = 256 mm × 256 mm, FOV = 256 mm; slice thickness = 2.0 mm, no gap, yielding a voxel resolution of 1 mm × 1 mm × 2 mm. Additional details have been previously described [6, 7].

### White matter hyperintensities segmentation

The Lesion Segmentation Tool (LST) version 1.2.3 in SPM12 was used to calculate the total volume of WMH [43]. This toolbox is open source and uses automated segmentation with high reliability. For lesion segmentation, LST seeds lesions based on spatial and intensity probabilities from T1 images and hyperintense outliers on T2 FLAIR images. The intracranial volume (ICV) was calculated using the “reverse brain masking” method [30]. Total WMH was then divided by ICV and multiplied by 100 to obtain a measure of lesion-to-cranial volume in percent units [6, 7, 43]. This measure served as the dependent variable in all analyses and was log-transformed to normalize its distribution, as required by the assumptions for ordinary least squares regression [45].

### Statistical analysis

Multiple linear regression was used to examine relationships between CRF, age, and WMH. We first fitted a model that investigated the relationship between age and WMH while controlling for APOE4, FH, body mass index, systolic blood pressure, and sex (Model 1). These covariates were applied to account for their contributions to interindividual variations in brain size, WMH, and/or risk for AD [1, 22, 41, 48].

Next, we refitted the original model while additionally including the OUES and age × OUES terms (Model 2). The OUES and age were centered at the mean of each variable. Where significant, the age × OUES term would indicate that the effect of age on WMH differs by CRF. A significant age × OUES interaction was further interrogated using simple main effects analyses. All analyses were conducted using IBM SPSS version 24. Statistical tests were considered significant at *p* < .05.

## Results

Similar to the larger WRAP cohort, many participants in this sample had a positive FH (71%) and were APOE4 positive (43%). The sample studied was 65.4% female. Other sample characteristics are presented in Table 1.

**Table 1:** Background characteristics

Characteristic	Value
Age (years)	64.19 (5.85) (49.58–74.96)
Female (%)	65.40
Education (years)	16.30 (2.35) (12–22)
FH (%)	71
APOE4 (%)	43
MMSE	29.37 (1.01) (24–30)
Hypertension (%)	14.0
Diabetes (%)	1.8
Smoker (%)	34.6
Beta blocker usage (%)	6.5
BMI (kg/m^2^)	27.84 (5.31) (17.65–48.03)
Systolic blood pressure (mmHg)	123.72 (15.66) (94–162)
Diastolic blood pressure (mmHg)	70.41 (9.62) (44–90)
OUES	1153.52 (290.72) (460–2290)
WMH (ml)	2.90 (5.23) (0.011–28.03)
ICV (ml)	1466.46 (140.65) (1175–1927)
Interval between MRI and GXT (years)	1.04 (1.04) (0–4.42)

All values presented as mean (standard deviation) (range) unless noted otherwise

*FH* family history of Alzheimer’s disease, *APOE4* apolipoprotein E ε4 allele carriage, *MMSE* Mini-Mental State Examination, *BMI* body mass index, *OUES* oxygen uptake efficiency slope, *WMH* white matter hyperintensities, *ICV* intracranial volume, *MRI* magnetic resonance imaging, *GXT* graded exercise testing

The results of Model 1 (see Table 2) revealed a strong positive association between age and WMH (*β*(SE) = .01 (.003); *t* = 3.89; *p* < .001). Sex was also a significant predictor of WMH (*β*(SE) = −.082 (.03); *t* = − 2.71, *p* = .008), with men harboring less WMH burden compared to women. Of note, APOE4 was not a significant predictor of WMH (*β*(SE) = −.015 (.031); *t* = − 0.50, *p* = .620). Similarly, FH was not a significant predictor of WMH (*β*(SE) = .029 (.034); *t* = 0.85, *p* = .396).

**Table 2:** Association between age and WMH

Variable	*β* (SE)	*t*	*p*
Age	.01(.003)	3.89	<.001
Sex	−.082 (.03)	−2.71	.008
SBP	.001 (.001)	0.35	.728
FH	.029 (.034)	0.85	.396
BMI	−.001 (.003)	−0.34	.731
APOE4	−.015 (.031)	−0.50	.620

*WMH* white matter hyperintensities, *SE* standard error, *SBP* systolic blood pressure, *FH* family history of Alzheimer’s disease, *BMI* body mass index, *APOE4* apolipoprotein E ε4 allele carriage

**Fig. 1:**
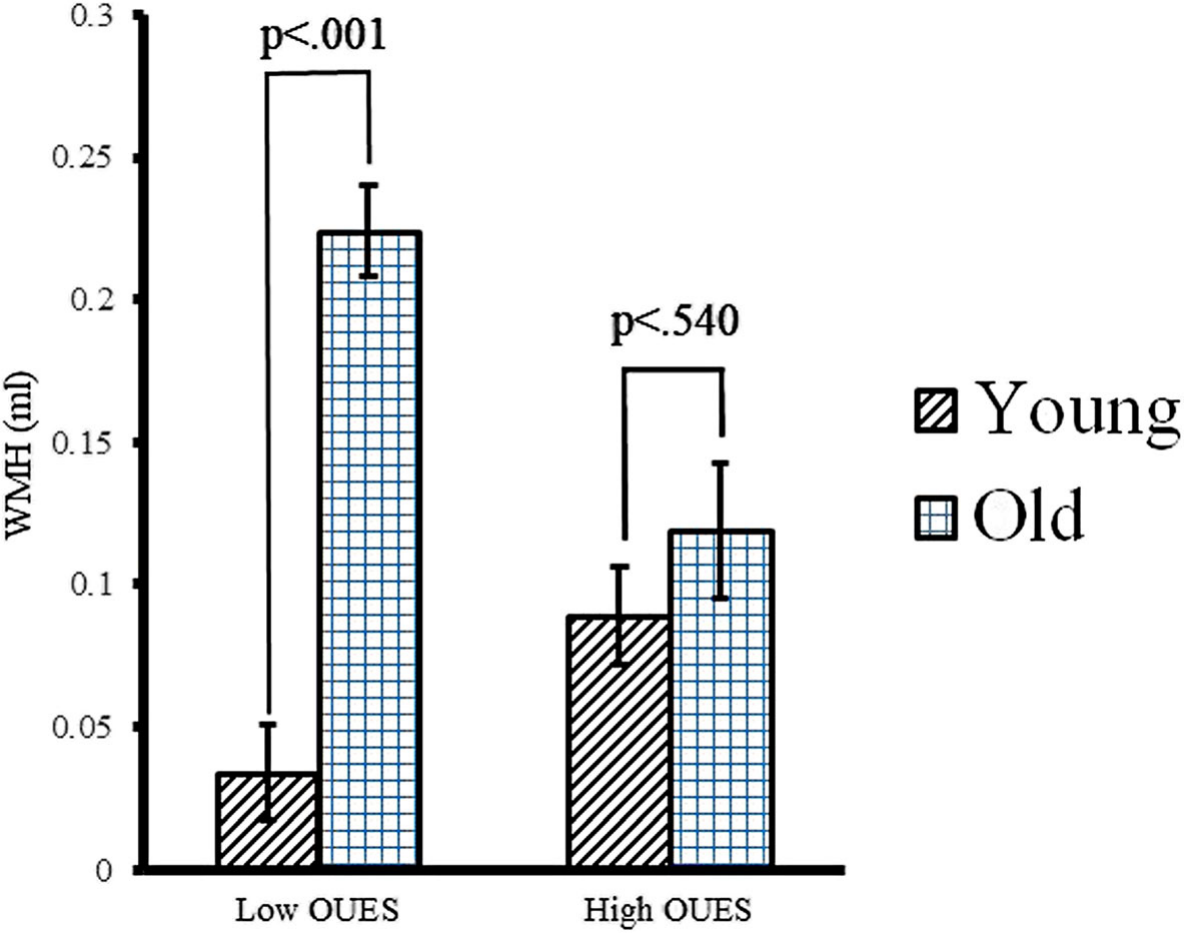
Estimated WMH as a function of age and OUES. Although age and OUES were modeled as continuous variables in our analyses, to depict these simple main effects, Low OUES vs High OUES were set to one standard deviation below vs above mean OUES. Similarly, Young vs Old were set to one standard deviation below vs above mean age WMH white matter hyperintensities, OUES oxygen uptake efficiency slope

Model 2 (see Table 3) showed a significant interaction between age and CRF on WMH (*β*(SE) = −.000024 (.0000096); *t* = − 2.47; *p* = .015). Per standard practice [12], we followed up on this interaction by conducting simple main effects analyses of the effect of age on WMH for Low OUES vs High OUES. To accomplish this, we set anchor points for Young vs Old and for Low OUES vs High OUES at one standard deviation below vs above the mean of each variable (see Table 1 for the respective values). As depicted in Fig. 1, these simple main effects analyses revealed that the effect of age on WMH accumulation remained significant in the Low OUES group (*β*(SE) = .19 (.043); *t* = 4.41; *p* < .001) but not in the High OUES group (*β*(SE) = .029 (.047); *t* = 0.62; *p* = .540).

**Table 3:** CRF attenuates the effect of age on WMH

Variable	*β* (SE)	*t*	*p*
Age	.009 (.003)	3.48	.001
Sex	−.073 (.035)	−2.07	.041
SBP	.000088 (.001)	0.091	.928
FH	.027 (.033)	0.81	.419
BMI	−.001 (.003)	−0.50	.619
APOE4	−.011 (.031)	−0.34	.732
OUES	−.000043 (.000063)	−0.68	.495
Age × OUES	−.000024 (.0000096)	−2.47	.015

CRF cardiorespiratory fitness, *WMH* white matter hyperintensities, *SE* standard error, *SBP* systolic blood pressure, *FH* family history of Alzheimer’s disease, *BMI* body mass index, *APOE4* apolipoprotein E ε4 allele carriage, *OUES* oxygen uptake efficiency slope

As noted earlier, our set of covariates (i.e., APOE4, FH, body mass index, systolic blood pressure) were selected based on prior evidence that they influence WMH and/or AD risk. However, in this study, none of these covariates were significantly associated with WMH at the .05 threshold (all *p* ≥ .124). Therefore, we repeated our analyses after excluding these covariates. Our original findings remained essentially unchanged. That is, there remained a positive association between age and WMH (*β*(SE) = .01 (.002); *t* = 4.3223; *p* < .001) and there remained a significant interaction between CRF and age on WMH (*β*(SE) = −.000025 (.000009); *t* = − 2.62; *p* = .01). For completeness sake, we opted to retain the original model that included the covariates.

Furthermore, we ran additional analyses to investigate whether our primary findings were influenced by potential confounders such as vascular risk factors (e.g., hypertension, smoking, and diabetes), beta blocker usage, and physical activity (as measured by caloric expenditure on the CHAMPS questionnaire [44]). The relationship between age and WMH remained significant when further adjusted for these covariates (*β*(SE) = .01 (.003); *t* = 3.23; *p* = .002). Similarly, the interaction between age and CRF on WMH also remained significant (*β*(SE) = −.000022 (.00001); *t* = − 2.22; *p* = .029). Accordingly, we opted to retain our original findings.

## Discussion

In this study, we found that older age was associated with greater accumulation of WMH. Importantly, our results showed that aerobic fitness attenuates the relationship between age and WMH. For those with low aerobic fitness, there was a significant difference in white matter lesion volume between younger and older participants. However, for those with high CRF, a similar deleterious effect of age on the prevalence of white matter lesions was not observed.

A prior study from our group reported that advancing age predisposes individuals to an aggregation of WMH, and that an increase in WMH is associated with decreased cognitive function [6]. Other groups have also found that WMH track with older age in the general population [15, 17, 31, 49]. Our results mirror these past findings despite the fact that our cohort is relatively younger. Of interest, we present novel results showing that CRF moderates the relationship between age and WMH. Given that WMH contributes to the clinical manifestation of AD [8, 16, 33, 40], CRF’s curtailment of WMH accumulation raises the possibility that CRF may, thereby, slow progression toward the clinical syndrome of AD.

Previous reports have found significant relationships between CRF and WMH using VO_2 peak_ as the index of CRF [11, 23, 50]. As discussed earlier, although deemed the gold standard for measuring CRF, true VO_2 peak_ is often unattainable by older adults. Hence, various alternatives have been considered in the literature such as non-GXT-based measures of CRF [7, 29] and OUES [3, 24]. Consistent with our recent publication [19], our present findings support the utility of the OUES as a viable metric for CRF in older adults, and highlight its sensitivity to important health outcomes such as cerebrovascular disease, brain aging, and risk for AD.

One possible mechanism for the results found in this study relates to cerebral perfusion. Previous studies in cognitively normal individuals have shown that an increase in WMH is associated with lower cerebral blood flow [9, 36]. Also, higher cardiorespiratory fitness is associated with increased cerebral blood flow and better cognition in older adults [9, 26]. Therefore, it is possible that higher cardiorespiratory fitness may protect against reductions in cerebral blood flow with advancing age, which then translates to a reduction in WMH. A formal test of this hypothesis will be the focus of future studies from our group.

This study is not without limitations. Neither APOE4 nor FH was significantly associated with WMH in our sample, even though other studies have previously reported such associations [5, 35]. Similarly, we did not assess relationships between WMH and β-amyloid or tau, the core pathological characteristics of AD, as such an investigation was not the objective of this study. Accordingly, we cannot definitively say that interindividual variations in WMH in this sample are AD specific. Our design is cross-sectional in nature, which limits our ability to draw causal inferences. Future studies incorporating longitudinal observations would provide clearer insights into the evolution of WMH over time and how CRF affects that trajectory. Also, because the WRAP cohort is largely composed of highly educated, non-Hispanic white individuals harboring specific risk factors for AD, there is a potential restriction of the generalizability of our results to the larger population. Relatedly, WRAP participants who volunteer for additional ancillary studies might differ in unmeasured ways from those who do not (e.g., our study sample was slightly older (mean age 64.19 years) than the larger WRAP cohort (mean age 62.89 years)). Lastly, we did not have information about the physical fitness of the participants earlier in their life, so we cannot determine how that may affect their current physical fitness or our outcomes of interest.

## Conclusion

We found that in a small sample at risk for AD, advancing age was associated with an accumulation of WMH. However, higher CRF attenuated this adverse impact of age on WMH. These findings contribute to the larger body of evidence highlighting the potential benefits of a physically active lifestyle, specifically as relates to improved cerebrovascular health and healthier brain aging.
